# TRIM72 promotes alveolar epithelial cell membrane repair and ameliorates lung fibrosis

**DOI:** 10.1186/s12931-020-01384-2

**Published:** 2020-05-29

**Authors:** Xiaofei Cong, Nagaraja Nagre, Jeremy Herrera, Andrew C. Pearson, Ian Pepper, Robell Morehouse, Hong-Long Ji, Dianhua Jiang, Rolf D. Hubmayr, Xiaoli Zhao

**Affiliations:** 1 Department of Physiological Sciences, Eastern Virginia Medical School, Norfolk, Virginia USA; 2 Department of Medicine, University of Minnesota, Minneapolis, MN USA; 3 Texas Lung Injury Institute, The University of Texas Health Science Center at Tyler, Tyler, TX USA; 4 Department of Medicine, Cedars Sinai Medical Center, Los Angeles, CA USA; 5 Division of Pulmonary and Critical Care Medicine, Mayo Clinic, Rochester, MN USA; 6 National Institute of General Medical Sciences, Bethesda, MD USA

**Keywords:** Idiopathic pulmonary fibrosis, Tripartite motif family protein 72, Alveolar epithelial cells, Membrane repair, Apoptosis

## Abstract

**Background:**

Chronic tissue injury was shown to induce progressive scarring in fibrotic diseases such as idiopathic pulmonary fibrosis (IPF), while an array of repair/regeneration and stress responses come to equilibrium to determine the outcome of injury at the organ level. In the lung, type I alveolar epithelial (ATI) cells constitute the epithelial barrier, while type II alveolar epithelial (ATII) cells play a pivotal role in regenerating the injured distal lungs. It had been demonstrated that eukaryotic cells possess repair machinery that can quickly patch the damaged plasma membrane after injury, and our previous studies discovered the membrane-mending role of Tripartite motif containing 72 (TRIM72) that expresses in a limited number of tissues including the lung. Nevertheless, the role of alveolar epithelial cell (AEC) repair in the pathogenesis of IPF has not been examined yet.

**Method:**

In this study, we tested the specific roles of TRIM72 in the repair of ATII cells and the development of lung fibrosis. The role of membrane repair was accessed by saponin assay on isolated primary ATII cells and rat ATII cell line. The anti-fibrotic potential of TRIM72 was tested with bleomycin-treated transgenic mice.

**Results:**

We showed that TRIM72 was upregulated following various injuries and in human IPF lungs. However, TRIM72 expression in ATII cells of the IPF lungs had aberrant subcellular localization. In vitro studies showed that TRIM72 repairs membrane injury of immortalized and primary ATIIs, leading to inhibition of stress-induced p53 activation and reduction in cell apoptosis. In vivo studies demonstrated that TRIM72 protects the integrity of the alveolar epithelial layer and reduces lung fibrosis.

**Conclusion:**

Our results suggest that TRIM72 protects injured lungs and ameliorates fibrosis through promoting post-injury repair of AECs.

## Introduction

Lung epithelial cells are frequently exposed to injuries by environmental hazards, cigarette smoking, free radicals, biotoxin, mechanical stresses, and hyperoxia, and plasma membrane disruptions often occur [1]. Eukaryotic cells possess the capacity to quickly restore the integrity of their plasma membrane [2], in order to minimize the need for energy- and time-consuming wounding healing and/or regeneration processes. Previous studies had revealed the mechanisms of plasma membrane repair in cells. Briefly, TRIM72 was discovered by an immunoproteomic screening of novel triad junction-enriched proteins in skeletal muscle cells [3]. Our previous studies suggest that TRIM72 is a critical component of the “membrane repair kit” in these cells [4, 5]. Under resting conditions, TRIM72 is associated with intracellular vesicles near the sarcolemma, possibly through its direct affinity to phosphatidylserine (PS) [5, 6]. Upon cell wounding, exposure to the extracellular oxidizing environment causes oligomerization of TRIM72 through disulfide bonds at C242 and leucine zipper homodimerization at L176/L183/L190/V197 [7]. The protein-vesicle complex traffics to plasma membrane wounds and forms repair patches [5]. In these capacities, TRIM72 functions as a sensor and an effector in the plasma membrane repair process. Follow-up studies show that TRIM72-mediated membrane repair is essential for skeletal muscle to resist exercise- or toxin-induced damages, and for the heart to endure ischemic reperfusion injuries (mainly through oxidative stress) [8–10]. Furthermore, studies in our lab revealed that TRIM72 mediates membrane repair in conjunction with Caveolin 1 and rescues the failing fate of type I alveolar epithelial cells (ATI) after stress, which in turn determines the severity of lung injuries following ventilator injury [11–13]. This suggests that cell injuries in different tissues caused by various injurious stimuli use similar repair mechanisms.

Idiopathic pulmonary fibrosis (IPF) is a fatal disease of progressive lung scarring with a median post-diagnosis life expectancy of fewer than 5 years. IPF posts an urgent medical need due to its poor prognosis and limited therapeutic options [14, 15]. Although the exact cause for IPF is unknown, the distinct feature of excessive epithelial cell loss and uncontrolled fibroblast activation suggests that it is a disease of epithelial injury and aberrant wound healing [16, 17]. Alveolar epithelial cells (AECs) are the direct targets of lung injury, and studies show that injury to AECs eventually leads to mesenchymal expansion and collagen deposition through epithelial-mesenchymal communication [1, 18]. Specifically, 90% of the alveolar surface is composed of long and thin ATI cells, while damages to ATI cells compromise the blood-gas barrier and exposes the underlying basement membrane and mesenchyme [19]. In addition, type II alveolar epithelial cells (ATII) were thought to play a pivotal role in the regeneration of ATI cells and pathogenesis of IPF based on the progenitor cell capacity of these cells and their ability to trans-differentiate into ATI cells [20, 21]. Indeed, IPF lungs show characteristic ATII cell hyperplasia and surfactant protein mutations were associated with susceptibility to IPF [22, 23]. Further studies show that regeneration of injured distal alveolar epithelium is critically determined by the “stemness” of ATII cells [24–26]. Consequently, ATII cell endoplasmic reticulum (ER) stress [27], senescence [28], or apoptosis [29] are all closely associated with the pathogenesis of IPF. Nevertheless, mechanisms and contribution of ATII membrane repair following injury in the development of lung fibrosis have not yet been examined.

In this study, we tested the membrane reparative role of TRIM72 in ATII cells and the role of TRIM72 in injury-induced lung fibrosis using an intratracheal bleomycin (bleo) injection (i.t.) model. Our results suggest that injury-induced upregulation of TRIM72 represents a protective mechanism against subsequent lung fibrosis while improving membrane repair of AEC cells and inhibiting the stress-activated p53 pathway are involved in the demonstrated effects of TRIM72. Thus, targeting TRIM72 may be a promising therapy for IPF.

## Methods

### Reagents

9-tert-Butyl Doxycycline (Dox) HCl was from Echelon Biosciences Inc. Bleo was from EMD Millipore. RNA isolation kit RNAeasy Mini was from Qiagen. All other reagents without description were from Sigma-Aldrich.

### Purification and in vitro administration of recombinant TRIM72 protein

Recombinant human TRIM72 protein (rhT72) was induced and purified as described before [13]. Briefly, the pMAL-c5X-hTRIM72 vector was used for the generation of rhT72 with an N-terminal maltose-binding protein (MBP) tag. It was transformed into high efficiency express competent *E. coli* (C2523, New England Biolabs). The recombinant protein was then produced and purified with AKTA prime protein purification system (GE Lifesciences). Yield and purity of recombinant protein were confirmed by SDS-PAGE and colloidal blue staining (LC6025, Invitrogen). Cultured cells in stretch assay plates were treated with an equal molar concentration of rhT72 or bovine serum albumin (BSA).

### Human lung samples

As described previously [30], aliquots of freshly frozen de-identified human lung tissues from histologically normal para-tumor areas (control) or pathologically confirmed IPF lungs were used for Western blot and immunostaining.

### Cell culture and lentiviral infection

Lentivirus production and transduction procedures were published before [12]. Briefly, human embryonic kidney (HEK)-293 T cells from ATCC (Catalog CRL-3216) were cultured in DMEM containing 10% FBS and 1% P/S until 80–90% confluence and transfected with L309-TRIM72 or L309 control vector, vesicular stomatitis virus G glycoprotein, Rev., and Rev. response element at 2:1:1:1 using Xfect reagent (Clontech). The rat ATII like epithelial cell line, RLE-6TN (ATCC, catalog CRL-2300), were cultured in F-12 K culture medium containing 10% FBS and 1% Pen/Strep (P/S). Cells were infected with L309-TRIM72 lentivirus or L309 control for 6 days, and then flow cytometry sorting of GFP fluorescence was performed to enrich GFP-positive cells.

### Animals

The generation of TRIM72 knockout (T72KO) and TRIM72 overexpressor (T72OE) mice had been described [11]. T72KO mice were backcrossed to C57BL/6 J (B6) background for at least 6 generations, and wild type (WT) B6 mice were used for control of the T72KO mice. Inducible T72OE heterozygous mice were on 129/B6 background, and WT littermate controls were used for these mice. Inducible T72OE mice were crossed with sftpc-eGFP mice [31] (No. 028356, Jackson lab) to generate inducible sftpc-eGFP/T72OE and sftpc-eGFP/WT littermate controls. To induce TRIM72 overexpression, Dox was administered to T72OE mice and WT littermates via intraperitoneal (i.p.) injection at a dose of 25 mg/kg body weight daily for consecutive 4 days before bleo i.t. injection. Dox injection continued for twice per week after bleo treatment. The efficiency of transgene induction by Dox i.p. was confirmed by Western blot (Fig. 7c). Control groups for bleo and HCl treatment were i.t. injected with an equal volume of PBS. Mice were housed in a sterile ventilated AAALAC-accredited animal facility at Eastern Virginia Medical School (EVMS). All mice were kept on a 12 h light/12 h dark cycle at 23 °C. Mice had ad libitum access to food and water. Mice of mixed gender were used for experiments, and no gender-based differences in phenotypes were identified in our study. The average age of mice was 2 ~ 6 months of age, and age-matched T72KO vs. B6 and T72OE vs. WT littermates were compared. All the experiments were approved by the Institutional Animal Care and Use Committee (IACUC) of the Eastern Virginia Medical School.

### Lung cell isolation from sftpc-eGFP mice

Dox was i.p. administered to mice for consecutive 2 days, and single-cell suspension from mouse lungs was made on day 3 as described previously [31]. Cells were suspended in PBS containing 1% FBS and 20 U/ml DNase. Flow cytometry and sorting of eGFP+ cells was performed using a BD FACSAria Fusion flow sorter (Becton Dickinson, Franklin Lakes, NJ) and analyzed using Flow Jo 10.1 software (Tree Star, Ashland, OR). 1 × 10^5^ eGFP+ cells from the sftpc-eGFP/T72OE or sftpc-eGFP/WT lungs were plated on 35 mm glass-bottom dish coated with 1:1 volume of 8–12 mg/ml Matrigel (E1270) and 3.5 mg/ml Collagen I (Corning, 354,236). The culture medium was 10% FBS, 1% P/S, 0.25 μg/ml amphotericin B, supplemented with insulin/transferrin/selenium in DMEM/F12K (Thermo Fisher Scientific). After 4 days of culture, the cells were treated with 2 μg/ml Dox for 48 h before membrane injury assays.

### Saponin injury of cells

Primary mouse ATII cells or RLE cells in culture were rinsed with calcium/magnesium-free PBS two times and placed on the sample stage of the Zeiss LSM 880 laser-scanning confocal microscope. Cells were then labeled with 2.5 μM FM4–64 membrane stain dye (Thermo Fisher Scientific) for 30–40 s, and PBS buffer containing 0.005% saponin plus 0.5 mM CaCl_2_ was added. Time-lapse videos of the GFP and FM4–6 fluorescence were taken at 2.5 s/image under Ex: 488 nm, Em: 507 nm, and Ex: 507 nm, Em: 723 nm, respectively. After recording, the region of interest (ROI) on individual cells was defined to quantify fluorescent intensity normalized to baseline (∆F/F0), and the raw data was analyzed and exported with Zen Black software (Zeiss).

### Stretching injury of cells

Stretch assay of RLE cells was performed previously [13]. 3 × 10^5^ RLE cells were plated on 6- well BioFlex dishes (170,404, FlexCell International) with collagen type I coating. Till about 100% density, cells in each well were washed with PBS and incubated with PBS buffer containing 0.5 mM CaCl_2_, 2.5 mg/ml fixable Fluorescein isothiocyanate-labeled dextran (FITC, D1820, Invitrogen) and 0.5 μM rhT72 protein or BSA for 2 min at room temperature. The cells were then stretched at 16% elongation with a duty cycle of 50% for 10 min. After stretching, cells sat for 5 min for membrane resealing to occur. Then cells were washed with PBS and incubated with fixable viability dye eFluor450 (11,000 in PBS, 65–0863, Thermo Fisher Scientific) for 5 min at RT to label the unrepaired cells. Then the cells were fixed with 4% paraformaldehyde (PFA) for 15 min at room temperature in the dark. After blocking with 5% goat serum and 0.3% TritonX-100 in PBS, immunostaining of p53 was performed overnight at 4 °C. After wash, cells were incubated with 1:400 Alexa Fluor 568 goat anti-mouse secondary antibody in blocking buffer. To show p53 nuclear localization, only 4, 6-diamidino-2-phenylindole (DAPI) and p53 were co-stained in another experiment. The cells were washed for 5 min 3 times, and images were captured on an IX73 inverted fluorescent microscope (Olympus) with DAPI (405 nm), FITC (488 nm), and a Cy3 (568 nm) filters. Ten images were randomly taken under 40 × for p53 and DAPI co-staining or 20 × for p53/FITC/eFluor co-staining. Number of eFluor-positive, FITC-positive, p53-positive, or double-positive cells were counted, injured cells = eFluor^+^ + FITC^+^ cells.

### Bleo injury of the cell

RLE cells were treated with 50 μg/ml bleo for 24 h before harvested for Western blot. For ubiquitination assay, 10 μM MG132 was added to the cells with or without bleo treatment 4 h before harvesting.

### Lung injury models

WT B6 mice were first anesthetized with 100 mg/kg ketamine and 10 mg/kg xylazine cocktail and received the following procedures to create models of lung injury:

### Mechanical ventilation

The procedures of low and high tidal volume ventilation were performed as previously reported [12]. Briefly, after anesthesia, mice were mechanically ventilated with room air and end-expiratory pressure of 3 cmH_2_O on a FlexiVent ventilator (SCIREQ, Montreal, QC, Canada) continuously for 3 h. A tidal volume of 30 ml/kg body weight at a rate of 60/min was used for injurious ventilation (IV), and 6 ml/kg body weight at a rate of 150/min was used for normal ventilation (NV). Lung tissues were harvested after 3 h of ventilation for RNA isolation or Western blot.

### HCl treatment

WT B6 mice received i.t. injection [32] of 50 μl 0.1 N hydrochloric acid (HCl) to create acid-induced lung injury. Lung tissues were harvested 24 h after HCl injection for RNA isolation or Western blot.

### Bleo model

To induce pulmonary fibrosis, mice were administered 75 μl sterile PBS or bleo at a dose of 1–2 U/kg body weight via i.t. injection. We found that i.t. injection efficacy can be significantly improved by increasing the aerosol injection volume from 50 to 75 μl, to position the microsprayer needle close to the trachea carina and to titrate the velocity of injection. Evans blue dye administrated this way showed broad dye distribution into all distal lung lobes (not shown). Mice used for detection of TRIM72 expression in various injurious models received 2 U/kg bleo; 2–3-month-old T72KO and WT B6 control received 1 U/kg bleo, and 5–6-month-old Dox-injected T72OE and WT littermate controls received 1.5 U/kg bleo; at day 0, day 3, day 7, day 14 or day 21, mice were euthanized, and the whole lung was dissected out. The right mainstem bronchus was tied off with 4–0 silk suture, and the right lung was cut and snap-frozen in liquid nitrogen. Right lung lobes were stored at − 80 °C for collagen content quantification using the hydroxyproline assay kit (Sigma-Aldrich, MAK008), Western blot, and total RNA isolation (RNeasy Mini Kit, QIAGEN, 74104). The left lung was inflated with 4% PFA at 20 cmH_2_O and fixed overnight at 4 °C. The left lobes were processed at the biorepository core of EVMS and used for H&E, Masson’s trichrome staining, and immunostaining. Injury score of a lung section from bleo treated lung was determined by T1α and was defined as follows: minimal (1), moderate (2), severe (3), maximum (4) disruption of alveolar epithelial integrity. BALF was obtained on day 3 after bleo i.t. with lavages using 1 ml PBS. Cells in BALF were counted with countess II FL automated cell counter (Thermo Fisher Scientific).

### Post-injury administration of rhT72 in bleo-treated mice

75 μl 1.5 U/kg body weight bleo (50% higher dose than that in B6 WT/T72KO experiment) were administered to the 8–12 weeks old B6 WT mice on day 0. Started on day 7, 50 μg rhT72 or equal molecular amount recombinant MBP protein was delivered via intraperitoneal injection to mice for 5 consecutive days. Mice were closely monitored for a total of 14 days. Mortality was recorded and lung samples were harvested for histology analysis. Kaplan-Meier survival curves were created for bleo-exposed rhT72- or recombinant MBP protein-treated mice.

### Hydroxyproline assay

To test the level of collagen in the mouse lungs, the right lungs were used for hydroxyproline assay (Sigma-Aldrich) according to the manufacturer’s procedure. Briefly, the right lungs were homogenized in PBS and hydrolyzed in 6 N HCl at 120 °C overnight. Diluted samples were incubated with 4-(dimethylamino) benzaldehyde (DMAB) for 90 min at 60 °C, and the oxidized hydroxyproline was determined at the absorbance of 560 nm.

### Western blot

Mouse lung samples were lysed in radioimmunoprecipitation assay (RIPA) buffer consisting of 10 mM Tris-HCl, 150 mM NaCl, 1 mM EDTA, 0.5 mM EGTA, 1% TritonX-100, 0.1% sodium deoxycholate, 0.1% SDS, and 1% protease inhibitor cocktail (Thermo Fisher Scientific) at 4 °C for 30 min. The lysates were centrifuged at 12,000 × RPM for 15 min at 4 °C. Protein concentrations in the supernatants were determined by the BCA protein assay kit (Bio-Rad). For Western blot, 40 μg of total protein were resolved by 10% SDS-PAGE followed by transferring to a 0.2 μm polyvinylidene fluoride (PVDF) membrane. The membrane was blocked with 5% non-fat milk in PBST (0.1% Tween-20) at room temperature for 30 min and then incubated with 5% milk or 5% BSA diluted primary antibody at 4 °C overnight. The membrane was then incubated with a 1:1000 diluted secondary antibody and detected with Pierce™ ECL Western Blot Substrate (Thermo Fisher Scientific). The following primary antibodies were used: rabbit anti-TRIM72 [11] (1014, 1:500, custom-made), rabbit anti-Phospho-p53 [33] (9284, 1:1000, Cell Signaling), mouse anti-p53 [33] (2524, 1:500 dilution, Cell Signaling), mouse anti-ubiquitin [34] (3936, 1:500, Cell signaling), and anti-mouse anti-β-actin [35] (1:5000, Sigma-Aldrich).

### Reverse transcription-quantitative polymerase chain reaction (RT-qPCR)

Total RNA from mouse right lung was extracted using the Qiagen RNAeasy Mini kit, following the manufacturer’s introduction. Concentrations and purity of RNA samples were determined using a Nanodrop LITE spectrophotometer (Thermo Fisher Scientific). Reverse transcription was performed with the ImProm-II™ Reverse Transcription System (Promega). Quantitative PCR was performed using 1 μl cDNA in a total volume of 10 μl containing 5 μl of 2 × SyberGreen PCR Master Mix (Life Technologies Corp.) and 0.2 μM gene-specific forward and reverse primers on a CFX96 Touch Real-Time PCR Detection System (Bio-Rad Laboratories). PCR condition was 36 cycles of 15 s at 95 °C and 1 min at 60 °C. Primer sequences were listed in Table 1. The specificity of all primers was verified by analysis of melting curves and agarose gel electrophoresis. The amplification efficiency of any single pair of primers was determined by analyzing the standard curve of serially diluted cDNA samples. The relative abundance of mRNA to Glyceraldehyde 3-phosphate dehydrogenase (*Gadph*) mRNA level in the same sample was calculated using the ^∆∆^Ct method [36].

**Table 1:** Nucleotide sequences of primers used for real-time PCR

Gene	Direction	Primer sequence	GenBank Accession #
*Trim72*	Forward Reverse	5′- CCGGCAAGGCTAGATATCCA − 3′ 5′- CTTCTGGTCTGAGCACTCCA − 3′	NM_001079932.3
*a-SMA*	Forward Reverse	5′- GCTGGTGATGATGCTCCCA − 3′ 5′- GCCCATTCCAACCATTACTCC − 3’	XM_006526606.2
*Fn1*	Forward Reverse	5’- GTGTAGCACAACTTCCAATTACGAA − 3′ 5′- GGAATTTCCGCCTCGAGTCT − 3’	XM_006495697.3
*Col1a1* *Cdh1* *Sftpc*	Forward Reverse	5’- CCAAGAAGACATCCCTGAAGTCA − 3′ 5′- TGCACGTCATCGCACACA − 3′	NM_007742.4
	Forward Reverse Forward Reverse	5′- CAGCCTTCTTTTCGGAAGACT − 3′ 5′- GGTAGACAGCTCCCTATGACTG − 3′	NM_009864.3
		5′- GAAGATGGCTCCAGAGAGCATC − 3′ 5′- GGACTCGGAACCAGTATCATGC − 3’	NM_011359.2
*Hopx*	Forward Reverse	5’- CAACAAGGTCAACAAGCACCC − 3′ 5′- GGCGCTGCTTAAACCATTTCT − 3’	NM_175606.3
*Aqp5*	Forward Reverse	5’- ATGAATCGGTTCAGCCCCTC − 3′ 5′- TCGATGGTCTTCTTCCGCTC − 3’	NM_009701.4
*Gadph*	Forward Reverse	5’- CGACTTCAACAGCAACTCCCACTCTTCC − 3′ 5′- TGGGTGGTCCAGGGTTTCTTACTCCTT − 3’	XM-017321385.1

*Trim72*, tripartite motif-containing protein 72; *a-SMA*, alpha-smooth muscle actin; *Fn1*, fibronectin-1; *Col1a1*, collagen, type 1, alpha 1; *Cdh1*, E-Cadherin; *Sftpc*, surfactant protein C; *Gapdh,* glyceraldehyde 3-phosphate dehydrogenase

### Immunostaining

Paraffin-embedded lung sections of 5 μm thin were deparaffinized, hydrated, and subjected to antigen retrieval. The following primary antibodies were used for immunostaining: mouse anti-α- smooth muscle actin (α-SMA, 1A4, Abcam), rabbit anti-surfactant protein C (SPC, sc-13,979, 1:50, Santa Cruz Biotechnology, Inc), rat anti-Pdpn (T1α, NBP2–03955, 1:200, Novus Biologicals), mouse anti-p53 (2524, 1:500, Cell Signaling Technology), rabbit anti-TRIM72 (ARP42971, 1:100, Aviva Systems Biology), cleaved caspase-3 (Alexa Fluor 488 conjugated, Asp 175, 1:50, Cell Signaling Technology) and mouse anti-human ATII cell membrane antigen (HT2–280, 1:100, Terrace Biotech). Fluorochrome-labeled species-specific secondary antibodies were used for immunofluorescence staining, and DAPI was used for nuclear staining. Stained sections were imaged using an Olympus IX73 fluorescent microscope. For competitive binding control for immunostaining on human lung sections, the mixture of HT2–280 antibody [37] and TRIM72 antibody [11] (10 μg/ml) was incubated with purified human recombinant TRIM72 (40 μg/ml) protein at a ratio of (1:4) before overnight primary antibody incubation at 4 °C. For quantification, 6 or more random images of each section were counted in a blinded fashion, and the number and percentage of positive cells were calculated and averaged. Three to six mice from each group were used for the experiment.

### Terminal deoxynucleotidyl transferase dUTP nick end-labeling (TUNEL) assay

Lung sections were assayed for alveolar cell apoptosis using the DeadEnd™ fluorometric TUNEL system (Promega Corp.), according to the manufacturer’s instruction. Rabbit anti-SPC were co-stained to identify the specificity of TUNEL-positive cells.

### Statistical analysis

Normality of continuous data was determined by the Anderson-Darling Normality Test Calculator. GraphPad Prism 7 was used for statistical analysis. Mann Whitney U test was used for the analysis of count data that do not follow a normal distribution pattern. Two-sided Student’s *t*-test was used to determine the statistical significance of the differences between two groups and one-way ANOVA with post hoc analysis was used to determine statistical significance among multiple groups. The Mantel-Cox Log Rank test was used to analyze the survival curve. A difference was considered statistically significant when *P* < 0.05. *P* values that are between 0.05 and 0.10 were labeled on an individual graph. All data were presented as mean ± standard error of the mean (SEM).

## Results

### TRIM72 expression in the lung is induced by various injurious stimuli

**Fig. 1:**
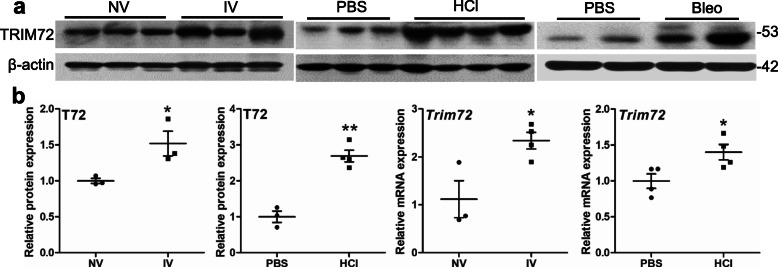
TRIM72 expression in the lung is induced by various injurious stimuli. **a** Western blot of TRIM72 protein in wildtype (WT) lungs received normal tidal volume (NV) or injurious ventilation (IV) for 3 h, intra-tracheal (i.t.) injection of PBS, 0.1 N hydrochloride acid (HCl) or 2 U/kg bleomycin (bleo). Tissues were harvested from mice 24 h after HCl i.t. or 14 d after bleo i.t.. The molecular weight (kDa) of proteins was labeled on all Western images; **b** quantification of TRIM72 protein or *Trim72* mRNA in IV vs. NV or HCl vs PBS lungs. Band intensity is normalized to β-actin and shown in mean ± SEM, *n* = 3 for NV and IV, n = 3 for PBS, *n* = 4 for HCl, *P < 0.05, **P < 0.01 compared to NV or PBS based on two-tailed student *t*-test

**Fig. 2:**
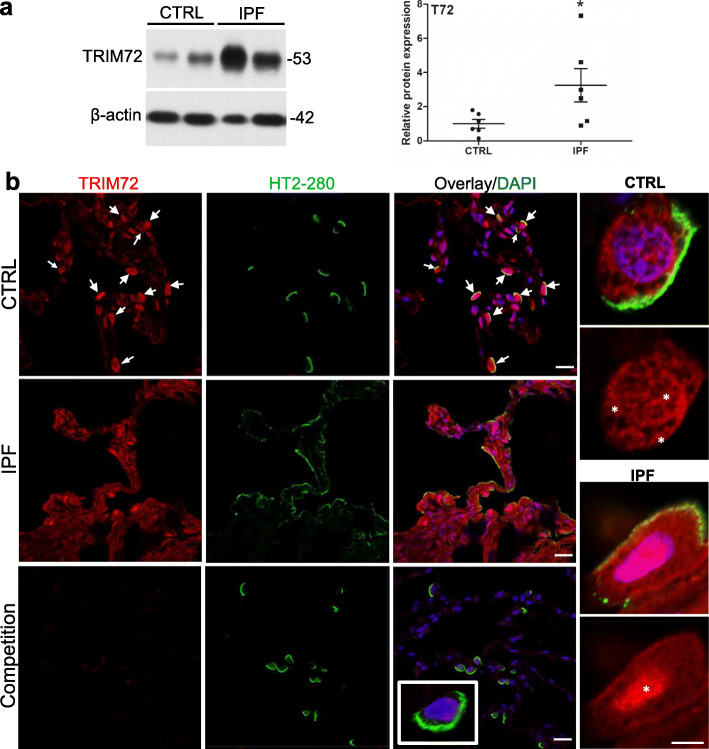
TRIM72 protein expression and distribution in the IPF lung. **a** Western blot and quantification of TRIM72 protein in histologically normal para-tumor (control, CTRL) human lung specimens and pathologically confirmed idiopathic pulmonary fibrosis (IPF) lung specimens. *n* = 6 for CTRL or IPF groups; **b** immunostaining of TRIM72 and HT2–280 on CTRL and IPF human lung sections. HT2–280 is a membrane-bound marker for human type II alveolar epithelial cells (ATII). Competitive immunostaining using 10 μg/ml recombinant human TRIM72 protein (rhT72) was included as a control for staining specificity of the anti-human TRIM72 antibody. White arrows = TRIM72 positive ATII cells; asterisks = cellular location of TRIM72. Scale bar = 20 μm for full images, = 5 μm for high magnification images

**Fig. 3:**
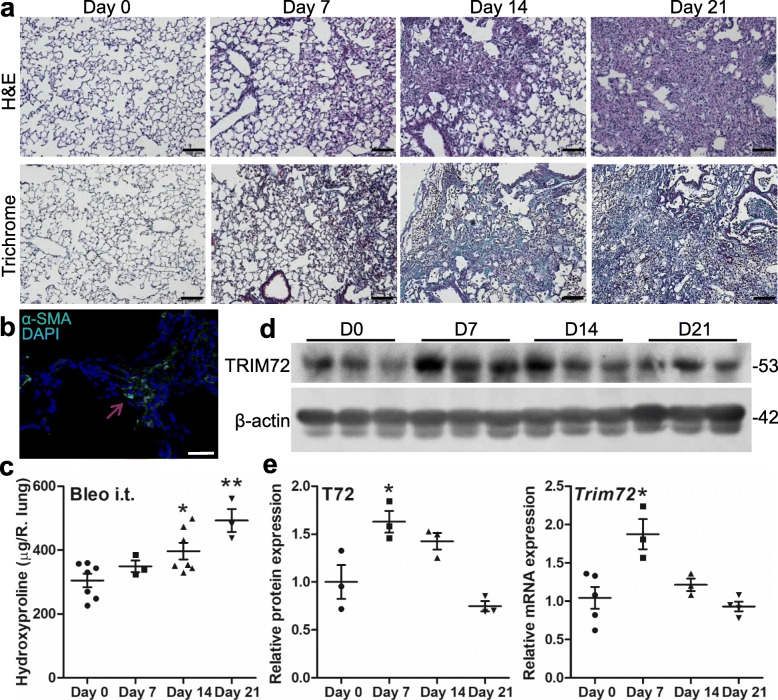
Tapering of bleomycin (bleo)-induced TRIM72 upregulation correlates with an increase in lung collagen content. **a** H&E and Masson’s trichrome staining on lung sections from 5 to 6-month-old B6 WT mice received i.t. of 75 μl 1.5 U/kg bleo. Tissues were harvested at days 0, 7, 14, and 21 after i.t. respectively. Scale bar = 100 μm; **b** α-smooth muscle actin immunostaining (α-SMA, green) on bleo-treated lung, counter-stained with DAPI (blue). Red arrow = fibrotic area. Scale bar = 50 μm; **c** hydroxyproline content per right lung after bleo i.t.; **d** Western blot of TRIM72 in bleo-treated WT lungs at the above time points; **e** quantification of TRIM72 protein and *Trim72* mRNA in mouse lung samples. *n* = 4 for D0 and D7, *n* = 6 for D14 and D21, *P < 0.05 or **P < 0.01 compared to day 0 based on one-way ANOVA with post hoc analysis

Endogenous reparative mechanisms first have to be able to sense and respond to injurious insults [38–40]. To examine TRIM72 expression following lung injury, we collected lung tissues subjected to 3 h of ventilation at 30 ml/kg tidal volume (IV), those received i.t. injection of 0.1 N HCl or 2 U/kg bleo [1]. Western results showed that all 3 injurious manipulations upregulated TRIM72 protein expression (Fig. 1a). Expression of *Trim72* mRNA also increased in the IV, HCl, or bleo-treated lung, compared to that in controls (Fig. 1b, 3e), suggesting that injury upregulates TRIM72 expression at the transcriptional level. We also measured TRIM72 expression in control and IPF human lung specimens. As shown in Fig. 2a, an increase in TRIM72 protein expression was seen in IPF as compared to the control lungs. Furthermore, TRIM72 expression in human ATII cells was detected by co-immunostaining of TRIM72 and HT2–280, a human ATII-specific membrane protein. Our results showed that TRIM72 expression was most abundant in HT2–280-positive ATII cells in human lungs and increased in IPF lungs (Fig. 2b). Enlarged images in Fig. 2b showed that the wide subcellular localization of TRIM72 in normal human ATII cells, i.e., plasma membrane, cytosol, and nucleus (stars), was changed into an aberrant nucleus-concentrated localization in the IPF ATII cells (Fig. 2b, star).

Furthermore, we performed bioinformatics mining on Harmonizome, which included 114 datasets [41]. We found that 1) microarray analysis detected increased TRIM72 expression in airway epithelial cells from patients with severe asthma [42]; 2) ATII cells from surfactant protein C (Sftpc)-deficient mice had increased TRIM72 expression [43]; 3) H5N1 viral infection increased TRIM72 expression in human airway epithelial Calu-3 cells (NCBI GEO dataset GSE43204); 4) expression of *Trim72* mRNA was upregulated in ATI and ATII cells from IPF lung through single-cell RNA-seq analysis (https://www.biorxiv.org/content/10.1101/759902v1). It is known that severe asthma and viral infection injure lung epithelial cells via excessive immune responses [1], while Sftpc-deficiency leads to endoplasmic reticulum stress in ATII cells [22] and cell susceptibility to mechanical forces due to increased surface tension. These data are summarized in Table 2. Thus, along with the pattern of TRIM72 upregulation in response to various injurious stimuli to the lung epithelium, the bioinformatics data suggests that TRIM72 is an injury responsive protein for a broad range of acute and chronic injuries to the lung.

**Table 2:** Expression analysis of lung TRIM72 in published datasets

Year	Author	Results	Model/sample resource	Reference No.
2014	N. Voraphani, et al.	Microarray analysis detected increased TRIM72 expression in airway epithelial cells from patients with severe asthma	Human asthma	[42]
2013	S. W. Glasser, et al.	ATII cells from surfactant protein C (Sftpc)-deficient mice had increased TRIM72 expression	Mouse LPS	[43]
2013	M. Katze, et al.	H5N1 viral infection increased TRIM72 expression in human airway epithelial Calu-3 cells	Human viral infection	NCBI GEO dataset GSE43204
2019	T. S. Adams, et al.	Expression of Trim72 mRNA was upregulated in ATI and ATII cells from IPF lung through single-cell RNA-seq analysis	Human IPF	Biorxiv.org/content/10.1101/759902v3

### Tapering of bleo-induced TRIM72 upregulation correlates with an increase in lung collagen

To examine the time course of injury-induced TRIM72 upregulation, we harvested lung tissues exposed to 1.5 U/kg bleo at various time points, i.e., day 0, 7, 14 and 21 after i.t. injection. Histology showed that bleo-treated WT lungs had a steady progression of alveolar structure disruption and expansion of scarred areas (Fig. 3a). The identity of fibrotic areas in bleo-treated lungs was confirmed by the detection of mesenchymal marker α-SMA amid the scarred lung areas (Fig. 3b) and a significant increase in the hydroxyproline content of the lung from day 14 to day 21 following bleo i.t. (Fig. 3c), suggesting the successful establishment of the bleo-induced lung injury and fibrosis model. Finally, Western blot showed that average TRIM72 protein and mRNA upregulation induced by bleo injury peaked at day 7 and gradually tapered off afterward (Fig. 3d, e). These data support that a single incidence of injury induces transitory TRIM72 upregulation, possibly as a compensatory protective mechanism, raising the intriguing possibility that IPF lungs had been exposed to repeated injuries and that TRIM72 upregulation in IPF lungs may have lost its protective capacity.

### TRIM72 promotes membrane repair of ATII cells

**Fig. 4:**
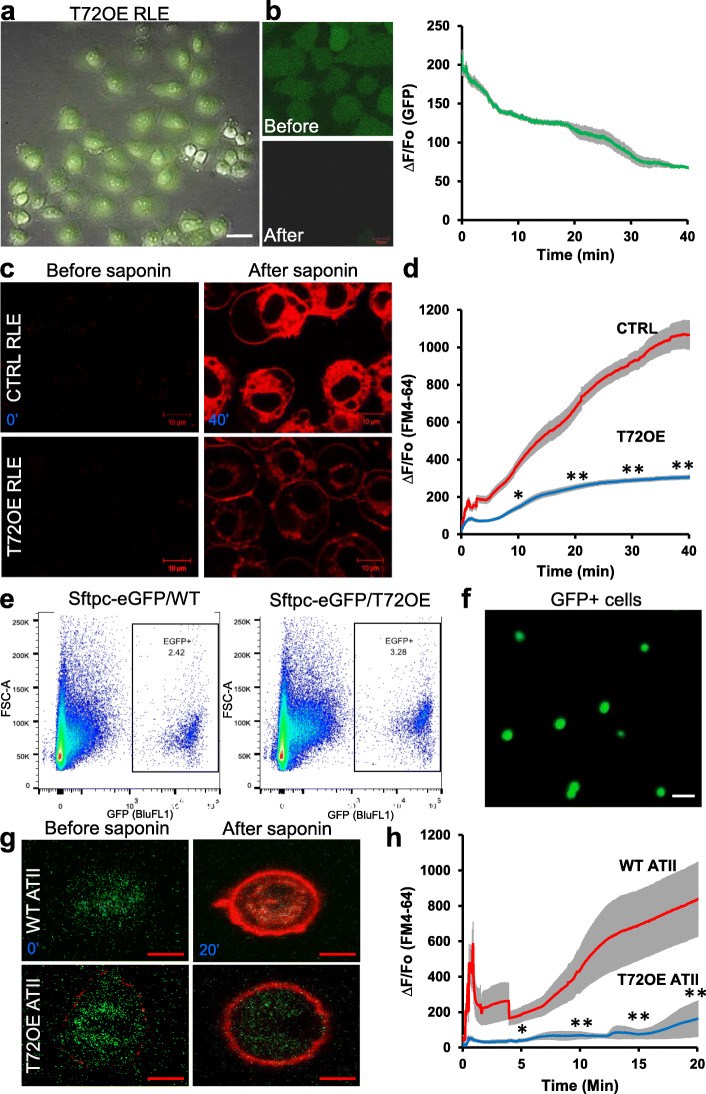
TRIM72 promotes membrane repair of ATII-like rat lung epithelial cells (RLE). **a** Lentivirus-mediated TRIM72 expression (T72OE) in RLE cells. Expression of GFP marker on the L309 vector indicates infected cells; **b** 0.005% saponin injury releases intracellular GFP from RLE cells; **c** representative images of FM4–64 dye entry in CTRL and T72OE RLE cells before (0′) and after saponin treatment (40′). Scale bar = 10 μm; **d** quantification of FM4–64 dye entry normalized to baseline fluorescence (∆F/F0). *n* = 36 cells for (CTRL RLE and *n* = 39 for T72OE RLE cells. *P < 0.05 or **P < 0.01 compared to WT at 10′, 20′, 30′ and 40′ based on two-sided student *t*-tests; **e** Flow sorting of GFP+ primary ATII cells from lungs of the sftpc-eGFP/WT and sftpc-eGFP/TRIM72 overexpressor (T72OE) mice; **f** representative image of freshly sorted GFP-positive primary ATII cells; scale bar = 50 μm; **g** representative images of FM4–64 dye entry in primary WT and T72OE ATII cells before (0′) and after saponin treatment (20′). Scale bar = 10 μm; **h** quantification of FM4–64 dye entry normalized to baseline fluorescence (∆F/F0). *n* = 8 cells for WT ATII and *n* = 4 for T72OE ATII cells. *P < 0.05, or **P < 0.01 compared to WT at 5′, 10′, 15′ and 20′ based on two-tailed student *t*-tests

To examine if TRIM72 acts in plasma membrane repair in ATII cells, we used ATII-like RLE with or without lentivirus-mediated TRIM72 expression (L309-T72). As shown in Fig. 4a, lentivirus infection has high efficiency, as indicated by the expression of GFP on the L309 vector. We showed that 0.005% saponin treatment releases intracellular GFP from RLE cells through porated plasma membrane (Fig. 4b). Furthermore, FM4–64 dye entry following saponin treatment was quantified in control and L309-T72 RLE cells. Our results showed that TRIM72 expression significantly reduced the degree and kinetics of FM4–64 entry into RLE cells (Fig. 4c-d), suggesting improved membrane repair. Next, we crossed the inducible T72OE [11, 44] male with the *Sftpc*-eGFP knock-in female [31] to establish the *sftpc*-EGFP/WT and *Sftpc*-EGFP/T72^OE^ mice. Flow cytometry sorting of eGFP positive cells was used to isolate primary ATII cells from these mice (Fig. 4e-f). Cells were then cultured on matrigel:collagen-coated dish for 5 days before membrane repair assays. Our results showed that T72OE significantly boosts the membrane repair capacity of primary ATII cells compared to WT cells, as indicated by FM4–64 entry (Fig. 4g-h). These data support that TRIM72 is an effective membrane repair machinery in ATII cells.

### TRIM72 salvages p53 activation in ATII cells

**Fig. 5:**
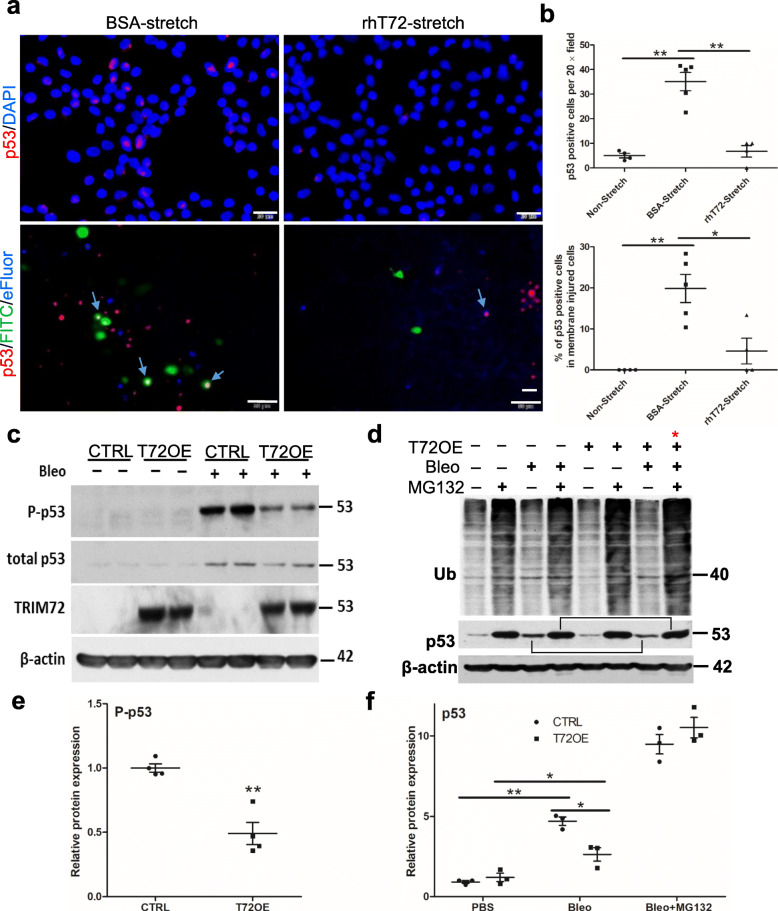
TRIM72 salvages stress-induced p53 activation in vitro. **a** Upper panels: immunostaining of total p53 (red) on stretch-injured RLE cells treated with bovine serum albumin (BSA) or rhT72; cells are counter-stained with DAPI (blue); lower panels: representative images of repaired cells labeled with FITC-dextran (green), non-repaired cells labeled with fixable cell vitality dye eFluor450 (blue) and post-fixation immunostaining of p53 (red) in these cells; blue arrows = p53+ FITC+ or p53 + eFluor+ cells; scale bar = 20 μm; **b** the number of p53 positive cells per 20 × field and quantification of p53+ cells among non-stretched and membrane injured cells (FITC+ plus eFluor+); **c** Western blot of Ser15 phosphorylated p53 (P-p53), total p53, and TRIM72 in CTRL or T72OE RLE cells with or without treatment of 50 μg/ml bleo; **b** Western blot detection of ubiquitin and total p53 in T72OE or CTRL, in the presence and absence of bleo and with or without MG132 to inhibit proteasome degradation of ubiquitinated substrates. Stars: Bleo+MG132-treated CTRL and T72OE RLE cells; brackets: bleo-induced total p53 with or without MG132 treatment; **c** and **d** quantification of P-p53 and p53 (in the absence and presence of MG132) from bleo-treated RLE cells. Relative protein expression of P-p53 or p53 was normalized to β-actin. n = 4, *P < 0.05, or **P < 0.01 based on two-tailed student *t*-test (P-p53) or one-way ANOVA with post hoc analysis (p53)

The p53 signaling pathway is a master sensor and effector of multiple stress stimuli [40]. It responds to cellular stresses and is turned on to modulate cell fates and fibrotic pathways [45–48]. A previous study showed that stretch injury induces p53 activation and apoptosis in vascular smooth muscle cells [49]. Here we confirmed the stretch-induced increase in p53 protein expression and nuclear translocation in RLE cells, while rhT72 significantly reduced p53 activation in stretched cells (Fig. 5a, b). Moreover, to assess p53 activation among membrane injured-cells, we applied FITC-dextran dye during stretching to label membrane injured and repaired cells and viability dye eFluor 450 after stretching to label non-repaired cells [13], and thus summarization of the two cell population represents total membrane injured-cells. Immunostaining of p53 was performed in fixed cells after stretching. Our data showed that p53 could be detected in cells that are negative for FITC or eFluor staining, suggesting that stretching stress can activate p53 in a small population of sensitive cells (< 1% of total cells) despite the lack of membrane injury (Fig. 5a-b). On the contrary, 20% of membrane injured cells (FITC+ plus eFluor+ cells) were positive for p53 (Fig. 5a-b), suggesting the presence of additional p53 activator during membrane injury. Exogenous rhT72 significantly inhibited the percentage of p53 positive cells among membrane injured cells (Fig. 5a-b), suggesting that TRIM72 modulates p53 activation after its activation by various stresses. To probe the mechanisms of TRIM72-mediated p53 inhibition, we examined Ser15 phosphorylation of p53 (p-p53) that is shown to retain p53 in the nucleus [50] and proteasome-mediated protein degradation that is the main regulatory pathway for p53 abundance. Our results showed that bleo treatment induced a remarkable increase in p-p53 and total p53 in control RLE cells while T72OE inhibited both of them (Fig. 5c-f). In addition, T72OE is shown to increase the overall ubiquitination activity in RLE cells (Fig. 5d, red stars) and that T72OE inhibition on bleo-induced p53 upregulation was largely neutralized by MG132 treatment to block proteasome degradation of ubiquitin-conjugated proteins (Fig. 5c-f). These results suggest that T72OE inhibits bleo induced-p53 activation via reducing its phosphorylation and promoting its proteasomal degradation.

### TRIM72 inhibits bleo-induced ATII cell apoptosis

**Fig. 6:**
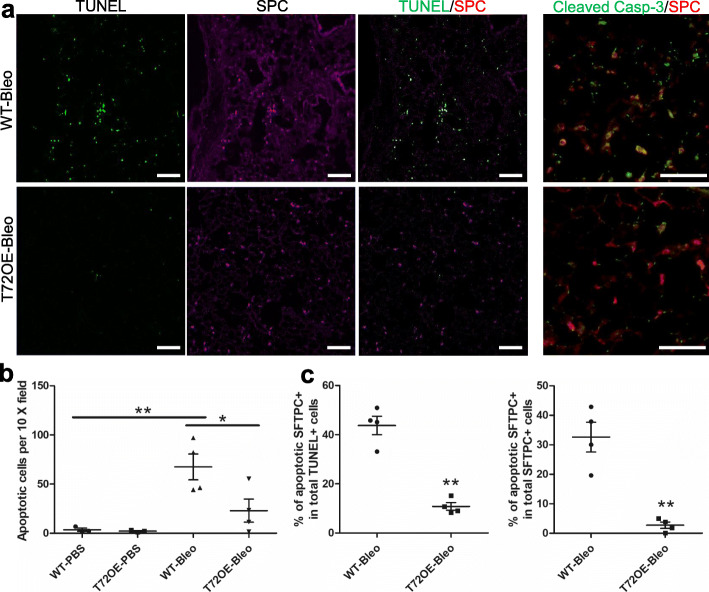
TRIM72 inhibits bleomycin (bleo)-induced ATII cell apoptosis. **a** Representative TUNEL, SFTPC, and cleaved caspase-3 (Casp-3) immunostaining on doxycycline (Dox)-injected bleo-treated WT and TRIM72 overexpressor (T72OE) lungs; scale bar = 100 μm; **b** quantification of the number of apoptotic cells (TUNEL+); **c** percentage of apoptotic ATII cells (SFTPC positive) among TUNEL positive cells, and apoptotic ATII cells among total ATII cells in PBS- or bleo-treated lungs; data = mean ± SEM, n = 3 for WT and T72OE PBS and *n* = 6 for WT and T72OE bleo groups, *P < 0.05 or **P < 0.01 compared to WT based on two-tailed student *t-*tests, or one-way ANOVA with post hoc analysis for comparison among different groups

Increased ATII cell death is a common feature of the IPF lungs and bleo-injured mouse lungs [29, 51]. Using WT and T72OE lungs received PBS or bleo i.t., we confirmed the significant increase in total apoptotic cells in bleo-treated WT lungs as compared to PBS-treated lungs by TUNEL assay and immunostaining of cleaved caspase-3 (Fig. 6a-b), among which over 40% apoptotic cells were also SPC positive (Fig. 6c), suggesting that ATII cell is a major target of bleo-induced cell apoptosis. In addition, over 30% of SPC-positive ATII cells were apoptotic (Fig. 6c), indicating the susceptibility of ATII cells to apoptosis. Compared to the WT lungs, T72OE lungs had a significant reduction in overall cell apoptosis and ATII apoptosis following bleo (Fig. 6b-c). This is consistent with our finding of TRIM72 inhibition on stress-induced p53 activation, which was shown to play a pro-apoptotic role in distressed cells if activated in excess [45, 52].

### TRIM72 maintains alveolar epithelial integrity in injured lungs

**Fig. 7:**
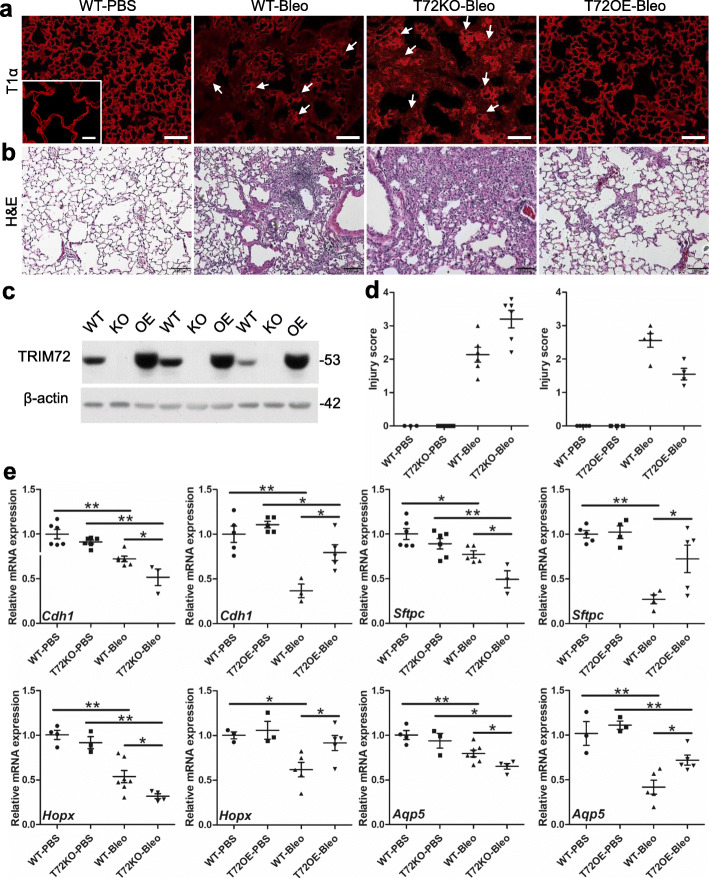
TRIM72 maintains alveolar epithelial integrity in stressed lungs. **a** Immunostaining of T1α to indicate alveolar epithelial integrity in PBS- and bleo-treated B6 WT, T72KO, and T72OE (Dox injected) lungs. Scale bar = 100 μm; **b** H&E staining of bleo-treated WT, T72KO, and T72OE lungs. Scale bar = 100 μm. The lungs from 2 to 3-month-old B6 WT and 5–6-month-old Dox-injected 129/B6 WT mice showed no difference in immunostaining of T1α or H&E staining; c. validation of experimental models. **a** Western blot shows good efficiency of Dox-induced TRIM72OE transgene induction and lack of TRIM72 expression in the TRIM72 knockout (KO) lungs; **d** injury scores based on T1α-staining indicated epithelial disruption. Mann Whitney U test was used since the injury scores are non-parametric data. The results indicated statistically significant differences; **e** relative mRNA expression levels of *Cdh1* (E-cadherin), *sftpc, Hopx,* and *Aqp5* in bleo-treated lungs, n = 4 for PBS groups and n = 6 for bleo groups; *P < 0.05 or **P < 0.01 compared to WT groups based on two-sided student *t*-tests

**Fig. 8:**
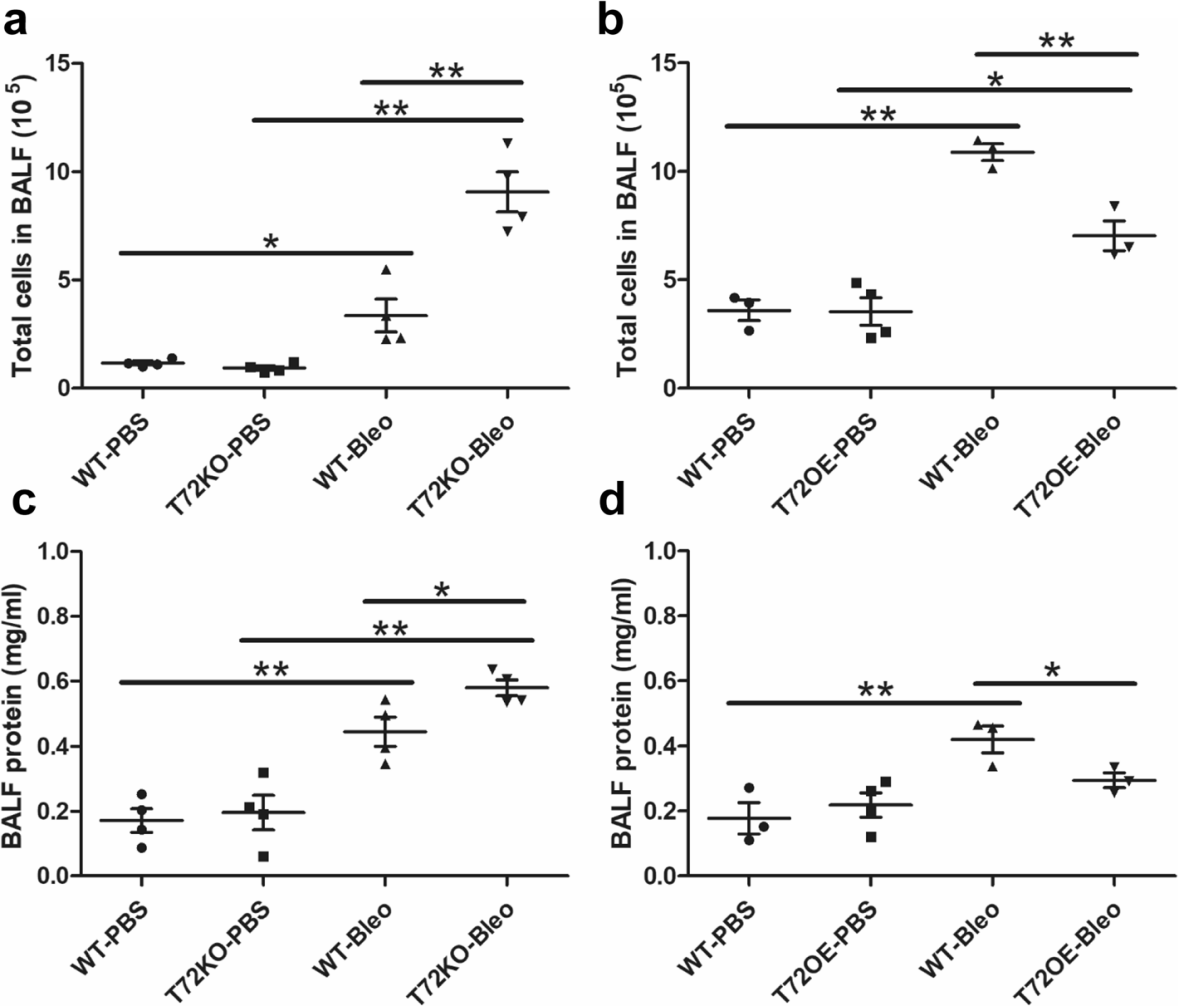
BAL fluid (BALF) cell profiles and BALF protein of bleo-injured lungs. **a** total BALF cells in WT vs. T72KO and **b** WT vs. T72OE lungs at day 3 after bleo i.t.; **c** BALF protein in WT vs. T72KO and **d** WT vs. T72OE lungs at day 3 after bleo i.t.. n = 4 for PBS and bleo groups, *P < 0.05 or **P < 0.01 based on one-way ANOVA with post hoc analysis

To examine if TRIM72 plays a significant protective effect on alveolar epithelial integrity due to its reparative role for both ATI and ATII cells, we performed T1α immunostaining in lungs. As shown in Fig. 7a, bleo-injury caused patchy disruption of T1α-positive alveolar epithelial layer in WT lungs, while in T72KO lungs, such disruption was wide-spread, and there were signs of epithelial thickening (Fig. 7a, arrows). On the contrary, the lungs of the T72OE mice only showed minor structural disruptions. A similar trend of histological injuries was seen in WT, T72KO, and T72OE lungs by H&E staining (Fig. 7b). Quantification of histological injury scores was done based on the extent of disrupted alveolar epithelial integrity based on T1α staining (Fig. 7d). In addition, we detected mRNA expressions of general epithelial marker *cdh1*, ATII cell marker *Sftpc*, ATI cell markers homeobox only protein x (*Hopx*) and aquaporin-5 (*Aqp5*) in bleo-injured lungs, and PBS controls (Fig. 7e). Our results showed that bleo-injured T72KO lungs had significantly lower levels of *Cdh1*, *Sftpc*, *Hopx,* and *Aqp5* mRNA as compared to the WT lungs, which were significantly ameliorated in the T72OE lungs (Fig. 7e). Furthermore, to assess barrier integrity and infiltration of inflammatory cells, we detected BAL fluid (BALF) protein and total cells in BALF in bleo-treated mice. Our results showed that T72KO lungs had increased lung permeability and cell infiltration in the lung, which was ameliorated in the T72OE lung, as compared with B6 WT controls or Dox-injected WT littermate, respectively (Fig. 8). These results suggest that TRIM72 reduces injury-induced epithelial disruption [24, 25].

### TRIM72 protects the lung from bleo-induced lung fibrosis

**Fig. 9:**
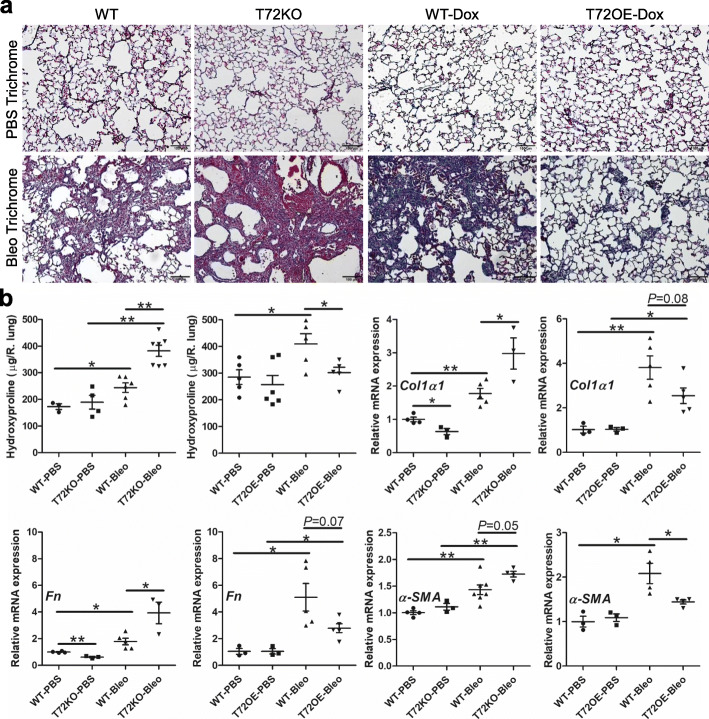
TRIM72 protects bleomycin (bleo)-induced lung injury and fibrosis. **a** Masson’s trichrome staining in bleo-treated B6 WT, T72KO, Dox-injected WT, and T72OE lungs. Based on the darkness of the blue stain from Trichrome staining, Dox-injected 5–6-month-old WT control had more collagen deposition than 2–3-month-old B6 WT control receiving PBS i.t. Scale bar = 100 μm; **b** hydroxyproline contents (normalized to WT-PBS controls), and relative mRNA expression of *α-SMA*, *Col1a1* (collagen 1 a1) and *Fn* (fibronectin) in bleo-treated B6 WT, T72KO, Dox-injected WT, and T72OE lungs. n = 4 for PBS groups and n = 6 for bleo groups, *P < 0.05, or **P < 0.01 based on one-way ANOVA with post hoc analysis

To further examine if TRIM72-mediated epithelial protection leads to reduced lung fibrosis, we assessed fibrotic markers in bleo-treated lungs at day 14 after bleo exposure. As compared to PBS-treated lungs, bleo-treated WT lungs had increased trichrome staining intensity, elevated hydroxyproline content as well as increased mRNA levels of extracellular matrix proteins Collagen I α1 (*Col1α1*) and Fibronectin (*Fn*) as well as mesenchymal marker *α-SMA* (Fig. 9). Trichrome staining showed that bleo-induced fibrosis was significantly worsened in T72KO lungs but ameliorated in the T72OE lungs compared to B6 WT controls or Dox-injected WT littermates (Fig. 9a). In addition, the T72KO lungs had increased hydroxyproline level, while the T72OE lungs had a significant reduction in hydroxyproline as compared to their appropriate WT controls (Fig. 9b). This is accompanied by an increase in the mRNA expressions of *Col1α1*, *Fn,* and *α-SMA* in the bleo-treated T72KO lungs and a decrease in expression of those 3 genes in the bleo-treated T72OE lungs compared to their controls (Fig. 9b). These data suggest that TRIM72 is indispensable for the protection against bleo-induced lung injury and fibrosis and that TRIM72 augmentation improves lung fibrosis.

**Fig. 10:**
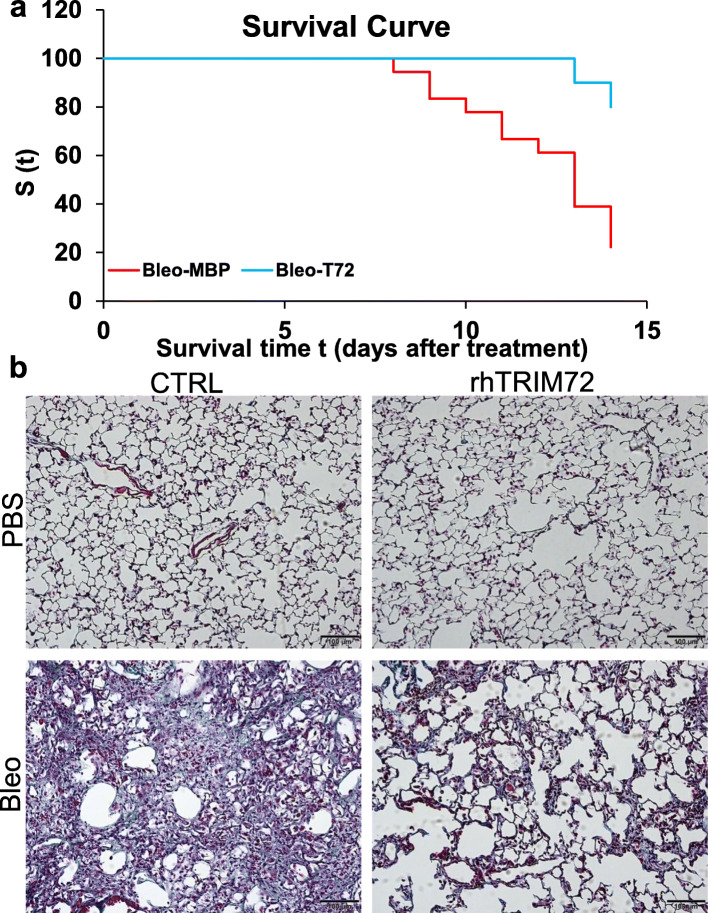
Post-injury delivery of recombinant human TRIM72 protein (rhT72) reduces mortality and prevents lung fibrosis in bleomycin (bleo)-exposed mice. **a** intraperitoneal injection of rhT72 at post-injury days 7–11 ameliorates the mortality in bleo-treated mice. Kaplan-Meier survival curves were created for bleo-exposed rhT72- or control protein-treated mice (*n* = 18 for MBP control group and = 10 for rhT72 group). Black arrow indicates the administration of rhT72 or CTRL protein. The Mantel-Cox Log Rank test yields a *p* = 0.0469; **b** Masson’s trichrome staining in 1.5 U/kg body weight bleo-treated B6 WT received CTRL or rhT72 protein at post-injury days 7–11. Scale bar = 100 μm

To address whether a repair therapy administrated after bleo exposure can mitigate lung fibrosis, we performed post-injury administration of rhT72. As shown in Fig. 10, intraperitoneal application of rhT72 post-bleo treatment at day 7–11 significantly attenuated mortality (Fig. 10a) and reduced Trichrome staining following bleo injury (Fig. 10b). The Mantel-Cox Log Rank test showed that the survival rate of the bleo/rhT72-treated mice was significantly higher than that of the bleo/CTRL-treated mice (*p* = 0.0469) (Fig. 10a). These data support the therapeutic value of rhT72 once injury-induced fibrosis has been established as seen in IPF patients.

## Discussion

In this study, we characterized the expression of a previously identified membrane repair protein, e.g., TRIM72, following various injurious stimuli to the lung and examined the role of TRIM72 in membrane repair of ATII cells. Furthermore, we assessed the consequences of genetic TRIM72 modulation in a mouse model of bleo-induced lung injury and fibrosis. Our data revealed an anti-injury and anti-fibrosis role of TRIM72, likely through promoting repair and survival of ATII cells and curtailing the stress-activated p53 pathway.

IPF is featured with excessive loss of alveolar epithelial cells and aberrant mesenchymal cell activation [51, 53], while direct and indirect evidence suggests that the possible causes of fibrosis are repeated injuries to the lung [16]. Specifically, 1) the IPF pathology of epithelial cell loss, regeneration, and fibroblast proliferation resembles that of skin wound healing following epidermal injury [54]; 2) risk factors for IPF such as virus infection, gastroesophageal reflux, radiation, cigarette smoke, and environmental exposures can all cause cell injury via a variety of mechanisms [1]; 3) genome-wide association studies identified susceptible variants for IPF [23, 55, 56] such as surfactant protein genes, mucin, telomerase and cytoskeleton genes, are also susceptible factors for increased cell injury and/or compromised cell repair [1]; 4) de novo injury to the lung such as mechanical ventilation has been shown to induce lung fibrosis in human patients [57]; 5) injurious maneuvers such as i.t. injection of bleo and HCl, or mechanical ventilation, recapitulate IPF pathology in animal models [58, 59]. To this end, tissue injury stimulates both post-injury stress and reparative responses, the equilibrium of which collectively determine the overall outcome of injury at organ level [39]. Among these reparative responses, our studies showed that membrane repair of wounded cells is a fundamental process to determine the split fate of acutely injured alveolar epithelial cells [1]. However, the role of alveolar epithelial cell membrane repair in the pathogenesis of IPF has not been investigated.

Harmful stress responses and reparative responses often manifest parallelly at the injured tissues through complex crosstalk, resembling the concurrent upregulation of pro-inflammatory and immuno-modulatory components of the immune system at pathogen-infected tissues. We reason that important reparative processes must be responsive to injurious stimuli. Our data in this study show that TRIM72 expression in the lung is upregulated in response to injurious ventilation, HCl instillation, and bleo exposure (Fig. 1). In addition, TRIM72 upregulation by injury is transient, and tapering of the TRIM72 level correlates with the rising of hydroxyproline level in the lung (Fig. 3). These findings suggest that lung tissues share similar compensatory cell protective mechanisms against various injurious insults, while post-injury responses are orchestrated in a temporally controlled fashion. Interestingly, the average TRIM72 level is elevated in IPF lungs (Fig. 2), suggesting that IPF lungs may be exposed to injurious insults. Nevertheless, different from the broad subcellular localization of TRIM72 in normal lung ATII cells where it is mostly concentrated in the lung, TRIM72 in ATII cells from IPF lung concentrates in the nucleus, raising the question whether the change in TRIM72 subcellular localization is a consequence of repeated lung injury and casting doubts on whether the upregulated TRIM72 in IPF lungs retains its physiological protective function.

Injury-sensitive reparative or stress responses need to be carefully dissected to differentiate these that are beneficial from those that are detrimental to eventual tissue fate. Through in vivo characterization of transgenic mouse lines of T72KO and T72OE, we found that ablation of TRIM72 reduces overall alveolar epithelial integrity, histological destruction, and barrier function of the lung following bleo injury while aggravates injury-induced inflammatory cell infiltration and lung fibrosis as compared to WT controls (Figs. 7, 8 and 9). We detected significant reductions in an overall epithelial marker *Cdh1*, ATI cell markers homeobox only protein x *Hopx* [60] and *Aqp5* [61], and ATII cell marker *sftpc* (Fig. 7e), suggesting that T72KO compromises repair responses of both ATI and ATII cells. This is consistent with our previous finding of the protective role of TRIM72 in ATI cells [11] and data in this study showing a membrane repair function on TRIM72 in ATII cells. On the other hand, TRIM72 overexpression protects the lung from bleo-induced lung injury and fibrosis (Fig. 7, 8 and 9). Importantly, post-injury administration of rhT72 reduces the mortality of bleo-exposed mice and weakens the Trichrome staining intensity of the lung (Fig. 10). Collectively, these data suggest that TRIM72 is a reparative molecule amid the post-injury stress responses in the lung. In addition, TRIM72 augmentation on top of endogenous upregulation in response to injurious stimuli provides further protection for lung injuries, suggesting that a membrane repair therapy may be beneficial for the progressive tissue destruction in IPF, which is thought to be a result of repeated micro-injuries [16].

Our previous studies suggest that TRIM72 plays an essential role in the repair of alveolar epithelial cells in particular ATI cells [1, 11, 12], which determine the extent of tissue pathology in a murine model of ventilator-induced lung injury. Exogenous rhT72 was shown to both improve cell repair and increase cell resilience to stretching wounding [11–13]. We found that an interaction between TRIM72 and Caveolin 1 (Cav1), a protein component of plasma membrane caveolae, and another membrane repair molecule [62–64], is particularly important for the membrane repair role of TRIM72 in ATI cells [12]. In this study, we confirmed that TRIM72 also expresses in human ATII cells (Fig. 2b), where Cav1 expression is thought to be missing [12, 65], which may be accountable for the largely plasma membrane localization of TRIM72 in ATI cells [11, 12] but the broader subcellular localization of TRIM72 in ATII cells. Nevertheless, using membrane repair assays [66], we showed that TRIM72 could improve the membrane repair capacity of immortalized and primary ATII cells (Fig. 4). Considering the close association of alveolar epithelial cell dysfunction with the pathogenesis of IPF [17, 18, 22, 67] and the progenitor role of ATII cells in regenerating the distal alveoli in adult lungs [24], the potential of TRIM72 for protecting against fibrosis in injured lungs is substantial due to the beneficial effect on both ATI and ATII cells. In this study, our data showed that T72OE reduces bleo-induced apoptosis of ATII cells (Fig. 6) and thus preserves the number of lung progenitor cells. As we show p53 inhibition upon T72OE or rhT72 treatment (Fig. 5), this is consistent with the reported role of the stress-activated p53 pathway in promoting apoptosis of distressed alveolar epithelial cells [47, 48]. In addition, as a previous report showing an inhibitory role of p53 activation on the self-renewal and proliferative capacities of club progenitor cells in the lung [68], it is possible that TRIM72 also inhibits the self-renewal capacity of ATII cells through inhibiting the p53 pathway, which may be addressed by future colony formation and lineage tracing studies. Consequentially, as the epithelial cell-specific increase in p53 was shown to aggravate both liver and pulmonary fibrosis [46–48], it is plausible that TRIM72 inhibition of p53 in alveolar epithelial cells reduces injury-induced lung fibrosis.

Studies reveal a complex crosstalk network of post-injury responses in the lung [69]. Epithelial cell injury was shown to trigger fibrogenesis via various mechanisms [70, 71], while epithelial sloughing following chronic injury destroys the basement membrane and alters extracellular matrix to prefer mesenchymal cell growth over the regenerating epithelium [72]. The p53 signaling pathway is a vital stress sensor in many cell types, which plays key roles in directing cell fate after injury or stress [40, 45, 73]. Our data show that stretching can trigger p53 activation in a small percentage of RLE cells despite the presence of membrane injury (p53 + FITC-eFluor- cells), while the presence of membrane injury causes p53 activation in about 20% of injured cells (Fig. 5a-b). Meanwhile, exogenous rhT72 significantly inhibited p53 activation in stretch-injured cells (Fig. 5). Overall, these findings suggest that stress is a direct activator of the p53 pathway, while TRIM72 act as a p53 modulator after its activation. Given the central role of p53 in directing cell fate and modulating fibrogenesis [74–76], we reason that TRIM72 reduces injury induced fibrosis both by improving membrane repair/cell vitality and inhibiting the stress pathway inhibition in alveolar epithelial cells. Furthermore, it is known that stress-induced post-translational modifications of p53 such as acetylation and phosphorylation of p53 disrupt the interaction between p53 and its E3 ubiquitin ligase Mdm2 and thus rescues the degradation of p53 protein [45]. In addition, p53 phosphorylation at Ser 15 was shown to facilitate the nuclear retention of p53 [50, 77], in addition to its interference of the p53/Mdm2 interaction. Our results showed that TRIM72 enhances the overall ubiquitination activity of RLE cells that is linked to bleo-induced p53 upregulation as well as Ser 15 phosphorylation of p53, suggesting that these post-translational modifications are the target mechanisms of TRIM72 modulation of p53, collectively leading to suppressed transcription of p53 target genes. Nevertheless, the mechanisms accounting for the enhanced alveolar epithelial cell p53 activation in bleo models and IPF [47, 48] are unclear where TRIM72 levels are increased. Our speculation is that since p53 is such a versatile sensor for multiple types of stresses, its final level of activation will be determined by the counterbalance of stress and p53 modulators such as TRIM72.

Another limitation of the study is inherent to the use of the bleo model for assessing lung injury and fibrosis, which is acute and reversible in nature as described in previous reports [16]. To improve the consistency of lung injury and fibrosis in this model, we conducted intratracheal injection of bleo aerosol into the distal lungs. In addition, we assessed main endpoints at day 14 after bleo injection to focus on the injury/fibrogenesis phase of this model, which is appropriate given that the proposed anti-fibrosis role of TRIM72 is based on its anti-injury property.

## Conclusion

In summary, our data revealed that TRIM72 repairs membrane injury of alveolar epithelial cells and inhibits post-injury activation of the p53 pathway. In vivo data revealed a cell/tissue-protective and anti-fibrosis effect of TRIM72 in injury-induced lung fibrosis model. Thus, targeting the membrane repair of alveolar epithelial cells and post-injury responses through TRIM72 represents a new direction for the development of IPF therapies.

## Data Availability

All relevant raw data are freely available upon request.

## Abbreviations

α-SMA: Alpha-smooth muscle actin
AEC: Alveolar epithelial cell
Aqp5: Aquaporin-5
ATI cells: Type I alveolar epithelial cells
ATII cells: Type II alveolar epithelial cells
B6: C57BL/6 J
BALF: BAL fluid
BSA: Bovine serum albumin
Cav1: Caveolin 1
Col1α1: Collagen I α1
DAPI: 4,6-diamidino-2-phenylindole
DMAB: 4-(dimethylamino) benzaldehyde
Dox: Doxycycline
ER: Endoplasmic reticulum
EVMS: Eastern Virginia Medical School
Fn: Fibronectin
Gadph: Glyceraldehyde 3-phosphate dehydrogenase
HCl: Hydrochloric acid
Hopx: Homeobox only protein x
IPF: Idiopathic pulmonary fibrosis
i.p: Intraperitoneal
i.t: Intratracheal
IV: Injurious ventilation
MBP: Maltose-binding protein
PFA: Paraformaldehyde
PS: Phosphatidylserine
PVDF: Polyvinylidene fluoride
P/S: Penicillin/streptomycin
rhT72: Recombinant human TRIM72
RIPA: Radioimmunoprecipitation assay
ROI: Region of interest
RT-qPCR: Reverse transcription-quantitative polymerase chain reaction
SEM: Standard error of the mean
SPC/*sftpc*: Surfactant protein C
T72KO: TRIM72 knockout
T72OE: TRIM72 overexpressor
TRIM72: Tripartite Motif Containing 72
TUNEL: Terminal deoxynucleotidyl transferase dUTP nick end-labeling
WT: Wildtype
